# A commentary on ‘Application of machine learning in surgery research: current uses and future directions – editorial’

**DOI:** 10.1097/JS9.0000000000000600

**Published:** 2023-07-18

**Authors:** Si-Un Frank Chiu, Chao-Ming Hung, Chong-Chi Chiu

**Affiliations:** a Department of Computer Science; b Department of Economics, Brown University, Providence, Rhode Island, USA; c Department of General Surgery; d Department of Medical Education and Research, E-Da Cancer Hospital; e School of Medicine, College of Medicine, I-Shou University, Kaohsiung, Taiwan

*Dear Editor*,

The editorial viewpoints by Sah *et al*.^1^ should raise widespread concerns among surgical professionals. In addition, we highlight more potential of machine learning (ML) in applying surgery research.

In the era of big data, the plethora of efforts toward gathering and analyzing patient data on a large scale is rapidly increasing. ML algorithms can assist surgeons in preoperative planning by analyzing a patient’s medical history, imaging data, and other relevant information. It can assess surgical risk more accurately and comprehensively than traditional methods. By exploring vast datasets encompassing various patient factors, such as demographics, comorbidities, and previous surgical outcomes, ML can identify patterns and risk factors that may not be evident to human observers. This information can aid surgeons in determining the optimal surgical approach, selecting appropriate interventions, and effectively managing patient risks accordingly.

The ability of ML allows for more precise surgical planning, reducing the likelihood of intraoperative surprises and improving overall surgical outcomes. Besides, ML algorithms have the potential to facilitate collaboration and knowledge sharing among surgeons and researchers^2^. ML can identify best practices and successful surgical techniques by analyzing large datasets and extracting patterns and insights. This information can be shared across surgical teams and institutions, promoting continuous learning and improvement in surgical procedures.

ML algorithms can contribute to the development of personalized medicine and precision surgery. ML can help identify the most effective treatment plans tailored to individual patients by analyzing patient-specific data, such as genetic information or imaging results. This personalized approach can improve surgical outcomes, reduce complications, and improve patient satisfaction. Moreover, ML can synergize with other emerging technologies, such as robotics and augmented reality, to further enhance surgical procedures. By integrating ML algorithms with robotic surgical systems, surgeons can benefit from real-time assistance, instant feedback, and precise guidance based on live data analysis, especially during complex emergency procedures^3^. Additionally, ML can contribute to developing augmented reality platforms, providing surgeons with valuable information overlayed in the surgical field, improving surgical accuracy, and reducing complications.

ML can play a significant role in continuous monitoring and postoperative care. ML algorithms can detect early signs of complications or postoperative infections by analyzing real-time patient data, such as vital signs or wearable device information. This algorithm enables timely interventions and proactive management, ultimately improving patient recovery and reducing readmission rates.

While some experts mention the ethical challenges of ML in surgery research, it is essential to highlight the potential positive impact on patient autonomy. ML algorithms can empower patients by providing personalized risk assessments, treatment options, and informed decision-making, which could promote patient engagement and autonomy in the surgical process, ensuring patients participate actively in their healthcare journeys. More importantly, ensuring data privacy and security is crucial because ML relies on vast patient data. Thus, robust measures must be in place to protect patient information and prevent unauthorized access. Transparent policies and ethical guidelines should govern the collection, storage, and use of patient data to maintain public trust and ensure the responsible application of ML in surgery research^4^.

ML algorithms could aid continuous quality improvement in surgical care. ML can identify trends, patterns, and areas for improvement by analyzing surgical outcomes, complications, and patient data. Experts could use this information to develop evidence-based guidelines, refine surgical techniques, and enhance overall surgical quality.

The integration of ML in surgery research allows for data-driven research and innovation. ML algorithms can process large datasets, uncover hidden correlations, and generate hypotheses for further investigation. This data-driven approach can lead to discoveries, advancements in surgical techniques, and the development of novel interventions, ultimately benefiting patient care.

The application of ML in surgery research can also lead to improved efficiency and resource allocation within healthcare systems^5^. Surgeons can allocate resources more effectively by accurately predicting postoperative outcomes, ensuring that high-risk patients receive the necessary attention and care. This optimization of resources can reduce costs and enhance the overall healthcare experience for patients.

In conclusion, the diverse applications of ML in surgery research present numerous benefits. However, we must carefully consider the challenges related to data standardization, interpretability, and ethical implications. With careful consideration of these challenges, ML has the potential to revolutionize surgical care, enabling personalized medicine, precision surgery, and improved patient autonomy. Continued advancements in ML algorithms, coupled with ongoing research and collaboration, will shape the future of surgery, leading to better outcomes and transforming the field for the benefit of patients worldwide.

## Ethical approval

This is only a commentary, not research involving patients. No ethical approval is required.

## Consent

This is only a commentary, not research involving patients. No patient consent is required.

## Sources of funding

This is a commentary. We have no funding for our commentary.

## Author contribution

S.-U.F.C.: conceptualization and writing the original draft; C.-M.H.: validation; C.-C.C.: conceptualization, supervision, submission, and correspondence.

## Conflicts of interest disclosure

There are no conflicts of interest in our commentary.

## Research registration unique identifying number (UIN)

This is a commentary to a study published in ‘International Journal of Surgery’. No UIN is required.

## Guarantor

Professor Chong-Chi Chiu.

## Data availability statement

This is only a commentary to a study published in ‘International Journal of Surgery’. There are no research data in our commentary.

## Provenance and peer review

This paper was not invited.
